# Bibliographical Notices

**Published:** 1847-10

**Authors:** 


### BIBLIOGRAPHICAL NOTICES.

*A System of Surgery, by J. M. Chelius, Doctor of Medicine and Surgery, Public professor of General and Ophthalmic Surgery, Director of the Chirurgical and Ophthalmic Clinic in the University of Heidelberg, &c. &c. &c. Translated from the German, and accompanied with additional Notes and Observations.* By John F. South, late Professor of Surgery to the Royal College of Surgeons of England, and one of the Surgeons to St. Thomas' Hospital. In three volumes, 8vo. Lea & Blanchard: Philadelphia, 1847.

The foundation of the present publication is one of those admirable *handbooks*, for which several recent German medical writers have rendered themselves famous. The object of the author in its preparation was to supply a text book which should give a short and clear description of Surgical Diseases and their treatment, and point out the best works on the several subjects. According to his plan, many things were slightly treated of, and others only hinted at, leaving the deficiencies to be supplied in his lectures. In subsequent editions, however, the author rendered his work more complete, and in the English translation Mr. South has supplied, very extensively, the omissions of the text.

In looking over the work, we have been particularly struck with the intimate acquaintance displayed with minute anatomy, and the changes and morbid products which occur from disease; the soundest views, both as to surgical pathology and treatment, are advanced on every subject, and generally in the clearest and most concise language. We meet with no affectation of originality; on the contrary, the best authorities are constantly referred to, and their very language quoted, where information or illustration is best attained in that way; we do not find the statements and opinions of others quoted, however, for the idle purpose of refutation and triumph, nor are the names of authors quietly omitted and shunned, lest the reader should discover the source from whence the most valuable materials are drawn. No such pettifogging is to be found in Chelius or his annotator; nor is it necessary for the interest of the work, for on every point treated of, evidence is afforded of a thorough acquaintance with the subject.

Diseases, according to Chelius, are either *dynamic* or *organic.* "This distinction can, however, only indicate a relatively predominant suffering of one or other phase of life, since the organic body presents in itself an entire whole, of which the several parts and phenomena are in the closest mutual connexion with each other.

"The organic diseases are especially those which originate in a destruction of the natural condition, form, and structure of organized tissues, and therefore may generally depend, 1. *On the disturbance of organic connexion;* 2. *On the unnatural union of parts;* 3. *On the presence of foreign bodies;* 4. *On the degeneration of organic parts, or on the production of new structures;* 5. *On the entire loss;* and, 6. *On the superfluity of organic parts.*"

"Organic diseases must be distinguished," according to our author, "into such as have their seat in parts inaccessible to mechanical contrivances, and to our organs of touch, and whose cure therefore can only be attempted by dietetic and pharmaceutic remedies; or those whose seat permits the employment of external means, and regulated contrivances, and which in most cases can be brought to heal only by these contrivances, with the assistance of dietetic and pharmaceutical aids. *We may therefore distinguish, as belonging to the province of Surgery, all those organic diseases which have their seat in parts accessible to our organs of touch, or which allow of the employment of mechanical means for their cure.*"

"Although inflammation is excluded from this general definition, we must, however, still enumerate it generally, and particularly among the manifold origins of surgical diseases, when it attacks external parts. Inflammation, in its course and results, produces for the most part organic changes, and requires, when attacking external parts, almost always the employment of the so-called surgical means; further, among the surgical diseases soon to be more particularly described, there is not one of which the cause is not inflammation, which in its course does not produce inflammation, or the cure of which is not, to a certain extent, simply and alone possible by inflammation."

The following is the author's division of the subjects embraced in his work.

1st Division.—*Of Inflammation*—general, peculiar, and of special organs.

2d Division.—*Diseases which consist in a disturbance of physical connexion.*—Solutions of continuity; *fresh*—wounds and fractures; *old solutions*—false joints, hare lip, &c., ulcers, fistula, &c. &c.

3d Division.—*Diseases dependant on unnatural adhesion of parts*—Anchylosis, adhesion and closure of natural openings, as the mouth, nose, rectum, vagina, &c. &c.

4th Division.—*Foreign bodies*—introduced externally into our organism; formed in our organism by the retention of natural products; accumulation of unnatural secreted fluids, from the concretion of secreted fluids.

5th Division.—*Diseases which consist in the degeneration of organic parts, or in the production of new structures*—Enlargement of Tongue, Clitoris, &c., Bronchocele, Warts, Bunions, &c.

6th Division.—*Loss of organic parts.*

7th Division.—*Superfluity of organic parts.*

8th Division.—*Display of the elementary management of Surgical operations.*

Prefixed, we have a notice of all the prominent works relating to surgery which have appeared from the earliest period of its literature, including Journals and Periodicals, the value of which can hardly be estimated by any one who has not occasion for extensive reference; but what is of more importance to most readers is a most complete analytical index at the end of the work, occupying upwards of seventy pages closely printed, in double column. It is the most perfect performance of the kind we have ever seen. We are informed, that the work has gone through six editions in Germany, and been translated into seven different languages—an evidence of general appreciation which few authors live, like Chelius, to see bestowed upon their works, but which all who examine this production will admit to be fully merited.

To Mr. South, the profession is certainly under very great obligations, not only for a good translation of an excellent work, but for the extensive and valuable additions he has made to it, and without which, the author's short notices of many subjects would have been unsatisfactory to either the student or practitioner. With the exception of Diseases of the Eye and Ear, which are omitted in consequence of the author having comprised them in a separate volume, this edition of the *Hand Book* of Chelius, with the notes and comments of Mr. South, and the references of Dr. Norris, may be regarded as the most comprehensive work on Surgery extant.

*Lectures on the Principles and Practice of Physic; delivered at King's College, London.* By THOMAS WATSON, M. D., Fellow of the Royal College of Physicians; late physician to the Middlesex Hospital; and formerly Fellow of St. John's College, Cambridge. *Third American, from the last London edition. Revised, with additions.* By D. FRANCIS CONDIE, M. D., Secretary of the College of Physicians; Author of a Treatise on Diseases of Children, &c. 8vo. pp. 101. Philadelphia: 1847.

*Handbuch der Medicinischen Klinik;* verfasst von Dr. CARL CANSTATT: königlich-bayerischem Gerichtsarzte und Mitgliede mehrerer gelehrter Gesellschaften. Zweite vermehrte Auflage. 8vo. Erster Band, S. 370: Dritter Band, S. 911. Vierter Band, S. 798. Erlangen, 1843.

*Lehrbuch der speciellen Nosologie und Therapie.* Von CONRAD HEINRICH FUCHS, Professor zu Göttingen. 8vo. Erster Band, S. 674. Göttingen, 1845: Zweiter Band, S. 1250: Göttingen, 1845–1847.

*Handbuch der Pathologie und Therapie.* Von Dr. C. A. WUNDERLICH, Professor der Medicin; z. z. Vorstand der medicinischen Klinik zu Tübingen. 8vo. Dritter Band, S. 616. Stuttgart, 1846–7.

There is no end to treatises on the Practice of Medicine. In our last number, we announced one from our own country and city; and we have heard of other *indigenous* productions, that are in course of preparation,—some positively at the full period of utero-gestation, and requiring but an enterprizing bibliopolical accoucheur to usher them into independent being,—either to die of *atelectasis*, or to attain full development amongst the prized varieties of the species.

To-day, we have to herald a few *exotics*—one of which has been transplanted into our own soil; and under the fostering cares that have been bestowed upon it, and still more, owing to its own intrinsic life-powers, has become naturalized, extensively known, and as extensively appreciated amongst us.

Of Dr. Watson's Lectures we have spoken more than once,—as often indeed as a new edition has appeared. We have now to announce a third. Of the original work we need not repeat the favourable sentiments to which we gave utterance when first we saw it. Without being complete, or equal in all its parts,—and where is the work that can exhibit entire *oneness* in its varied details?—it is generally excellent in its descriptions, didactic, and well expressed. There is something, too, attractive to the young more especially in the form and style of "Lectures." The reader seems to feel that he is listening to, rather than reading, the discourse; and more interest is felt, by the young more especially, and less fatigue experienced, than in more profound and serious essays on the same subjects, not conveyed in the like manner.

The basis—*Grundtext*—of Watson is the same as in the last American edition. The notes of the American editor, contained in that edition, are equally in this; but in addition we notice others, which are good and appropriate. We know, indeed, of no one to whom the task of editing such a work could have been committed with more propriety than to Dr. Condie. Possessed of a knowledge of various languages—Teutonic and Romanic—zealous in maintaining himself *a porteè* with the existing condition of medical science everywhere; judicious and impartial; a supporter of no exclusive sect, system, school or *clique;* cosmopolitan in science, as every one ought to be,—it is a disgrace, indeed, to him that is not,—he is well adapted to be the exponent of the existing state of medical knowledge here and elsewhere. As the editor of a work like the one before us, he has not room enough to express his opinions fully and freely, but where he has done so, his additions have been of such a character as to cause us to regret that more *paper-room* had not been allowed him.

The "*Manual of Clinical Medicine*" of Dr. Canstatt is anything but a "Manual" (*Handbuch:*) as well might we term a blunderbuss a pocket pistol. It is not yet complete; and as the able author is dead, doubts may be entertained whether it ever will be. We have recently heard, however, from Germany, that another volume is about to appear. Both it and the "*Handbuch*" of M. Wunderlich are examples of what we took occasion to deplore, in noticing certain German works a short time ago.

Instead of issuing the volumes in proper order, we, at times—as in the case of that of M. Wunderlich—receive first the *third* volume. M. Canstatt's work—which is now in its second edition—is on a good plan. The first volume, which, in this case, *did* appear first, is on the elementary forms of disease, or what he terms "the morphological part of clinical medicine"—*Morphologischer Theil der Klinik;* and embraces Hypertrophy, Atrophy, Plethora, Anæmia, Chlorosis, Hyperæmia, Inflammation, Hemorrhage, Blood Disease, Anomaly of Secretions, Dropsy, Bright's Disease, Pneumatosis, Adiposis, Homologous Transformations of Tissue, (*Homoöplasie*,) Carcinoma, Tuberculosis and Scrophulosis, Lithiasis, Invermination, Softening, Induration, Fever, Neuroses, Nervous Erethism, (*Algien*,) Spasm, Anæsthesia, Acinesia, and Psychoses,—all subjects which he deemed it advisable to treat in a general manner, before passing to Special Pathology and Therapeutics, which occupy the remaining volumes. The *division*, which he adopts in them, is the anatomical: the third volume, for instance, which was issued after the first—the second not having appeared—embraces diseases of the head, spinal marrow, nerves and air-passages. In the detail of each of these, there is much system, and an ample bibliography, with numerous references to authorities, in the practical portions more especially. In this respect it differs greatly from the work next in order.

Of the work of Professor Fuchs of Göttingen we have received the first and second volumes,—the whole being intended to be comprised in that number; but it certainly is not complete. There are many topics that ought still to be treated of.

We confess we are not—as utilitarians—so well pleased with this production as with the others. It has not the same *practical* air about it as they have. We say "*practical*"—not in the cant language of the day, which is too apt to regard everything not practical that does not treat of *dosing*, but it seems to us too much attention is given by M. Fuchs to nosology, and to endless divisions and subdivisions, so that in the absence of an Index, which the parts issued do not possess, it is a task of no little labour to find out any special topic on which we may desire to see the sentiments of the author. The first volume, which is addressed to his "beloved preceptor"—*seinem geliebten Lehrer*, Dr. J. L. Schönlein—a highly esteemed name in his *Vaterland*—treats of the Classes and Families of Disease, to each of which he gives learned appellations derived from the Greek, and for which he will have, of course, the blessings of the "harmless drudge"—the lexicographer; for we doubt whether they will ever be extensively disseminated except by him. We shall only refer here to his classes,—the arrangement of which he follows in his second volume, in inquiring into the genera and species—"*Gattungen und Arten.*" These are: *First.* "Diseases of blood-life"—*Hæmatonosen. Second.* "Diseases of nerve-life"—*Neuronosen;* and *Third.* "Diseases of Form and Formation"—*Morphonosen.* Of each of these he has several orders, families, genera and species, leading—as we have said—to learned confusion; and exhibiting much and useless metaphysical subtlety. The author is, however, a man of decided learning, and eminently instructed in his profession. His book will doubtless be properly appreciated among his countrymen, and may find, with advantage, a place in the library of every one acquainted with German Medical Literature.

The last of the books whose titles are at the head of this article, is the most adapted to our taste, both by its arrangement, and the fulfilment of details. It adopts the best of all divisions—that according to organs or apparatuses. The *third* volume, which is the *first* to appear—and the only one, by the way, that *has* appeared—embraces the diseases of the circulatory and respiratory organs, test subjects for an author, and one on which he cannot write a respectable treatise without being instructed in all modern diagnostic methods. The Prospectus states, that the third volume will embrace the diseases mentioned, as well as those of the digestive and urinary organs; but the volume really concludes with those of the respiratory organs. The whole work is promised by Easter, 1848. Its author is certainly a well read, learned and sensible physician; and his work is an excellent dissertation on the subjects comprised in it. We have consulted it on numerous occasions with much pleasure and profit.

*The Dispensatory of the United States of America.* By GEORGE B. WOOD, M. D., Professor of Materia Medica and Pharmacy in the University of Pennsylvania, one of the Physicians of the Pennsylvania Hospital, etc., etc., and FRANKLIN BACHE, M.D., Professor of Chemistry in Jefferson Medical College of Philadelphia, one of the Vice Presidents of the American Philosophical Society, etc., etc. *Seventh Edition, carefully Revised.* Grigg, Elliot & Co. Philadelphia: 1847.

We have much pleasure in announcing a new edition of this excellent work; not because of any deficiencies in the former edition, or of any large additions to be expected in the present, derived from improvements made within the brief period which has elapsed, but as evidence of a just appreciation of its merits. The authors have carefully gleaned from the periodical journals, and recent European treatises, everything of value which came within the scope of the work, so that it is fully up to the day. The publishers, too, have done justice to the work by the style in which it is brought out.

---

*Wood's Quarterly Retrospect of American and Foreign Practical Medicine and Surgery.* April to July, 1847. No. 1, Vol. 1. Richard and George S. Wood: New York.

This is a new publication, just projected, on the plan of Braithwaite and Ranking, but instead of semi-annually, it is to appear quarterly.

The present number contains sixty-four pages, double column, of well selected matter, embracing sixty-nine American articles and forty-one Foreign. The fault we would find with it is the want of attention to typographical accuracy.

We can see no reason why such a publication of indigenous origin should not succeed as well as the exotics, which we believe thrive bravely. It is perhaps true now, as in ancient days, that a prophet hath honour in all countries save his own. We see evidences of it daily; and yet as a people we are charged with being egotistical and self-sufficient beyond all example. We certainly are a little boastful, and have a right to be; we have more to be proud of than any other nation, as it regards our country and its institutions, and all that is wanting to perfect our national character is a national literature. Why should not American learning, American talents and enterprise in literature and authorship, be fostered and protected as well as the grosser arts? Individually, we are the richest people on the earth; but the wealth that we have will only serve to brutalize and degrade us, without the refining influences of science and the arts.

